# Obstructive sleep apnea is associated with increased QT corrected interval dispersion: the effects of continuous positive airway pressure^☆^

**DOI:** 10.1016/j.bjorl.2017.03.005

**Published:** 2017-03-31

**Authors:** Nagihan Bilal, Nursel Dikmen, Fulsen Bozkus, Aylin Sungur, Selman Sarica, Israfil Orhan, Anil Samur

**Affiliations:** a Kahramanmaras Sutcu Imam University, Faculty of Medicine, Department of Otorhinolaryngology, Kahramanmaras, Turkey; b Kahramanmaras Necip Fazil City Hospital, Department of Chest Disease, Kahramanmaras, Turkey; c Kahramanmaras Sutcu Imam University, Faculty of Medicine, Department of Chest Disease, Kahramanmaras, Turkey; d Kahramanmaras Necip Fazil City Hospital, Department of Cardiology, Kahramanmaras, Turkey; e Akdeniz University, Faculty of Medicine, Department of Biostatistics and Medical Informatics, Antalya, Turkey

**Keywords:** QTcd, Arrhythmia, Obstructive sleep apnea, Apnea hypopnea index, QTcd, Arritmia, Apneia obstrutiva do sono, Índice de apneia-hipopneia

## Abstract

**Introduction:**

Severe obstructive sleep apnea is associated with increased QT corrected interval dispersion and continuous positive airway pressure is thought to improve this arrhythmogenic marker.

**Objective:**

The aim of the study was to determine the decrease of ratio of cardiovascular risk in patients with obstructive sleep apnea.

**Methods:**

The study included 65 patients with severe obstructive sleep apnea who had an apnea-hypopnea index score of >30. Each patient underwent 12-channel electrocardiogram monitoring and polysomnography. Patients with an apnea-hypopnea index score of <5 were used as the control group. The control group also underwent electrocardiogram monitoring and polysomnography testing. The QT corrected interval dispersion levels of both groups were calculated. Three months after continuous positive airway pressure treatment, electrocardiogram recordings were obtained from the 65 patients with severe obstructive sleep apnea again, and their QT corrected interval dispersion values were calculated.

**Results:**

There were 44 male and 21 female patients with severe obstructive sleep apnea syndrome. The age, gender, body mass index, initial saturation, minimum saturation, average saturation, and desaturation index were determined in both groups. The QT corrected intervals of the obstructive sleep apnea patients (62.48 ± 16.29 ms) were significantly higher (*p* = 0.001) than those of the control group (29.72 ± 6.30 ms). There were statistically significant differences between the QT corrected values before and after the continuous positive airway pressure treatment, with pretreatment QT corrected intervals of 62.48 ± 16.29 ms and 3-month post-treatment values of 41.42 ± 16.96 ms (*p* = 0.001). There was a positive and significant correlation between QT corrected interval dispersion periods and the apnea-hypopnea index and hypopnea index in obstructive sleep apnea patients (*p* = 0.001; *r* = 0.71; *p* = 0.001; *r* = 0.679, respectively).

**Conclusion:**

Continuous positive airway pressure treatment reduced the QT corrected interval dispersion in patients with severe obstructive sleep apnea. In addition, shortening the QT corrected interval dispersion periods in patients with severe obstructive sleep apnea may reduce their risk of arrhythmias and cardiovascular disease.

## Resumo

**Introdução:**

A apneia obstrutiva do sono grave está associada a uma maior dispersão do intervalo QT corrigido e acredita-se que a pressão positiva contínua nas vias aéreas melhore esse marcador arritmogênico.

**Objetivo:**

Determinar a diminuição da razão de risco cardiovascular em pacientes com apneia obstrutiva do sono.

**Método:**

O estudo incluiu 65 pacientes com apneia obstrutiva do sono grave que apresentavam índice de apneia-hipopneia > 30. Cada paciente foi submetido à monitorização por eletrocardiograma de 12 derivações e polissonografia. Os pacientes com escore de índice de apneia-hipopneia < 5 foram utilizados como o grupo de controle. O grupo de controle também foi submetido à monitorização por eletrocardiograma e teste de polissonografia. Os níveis de dispersão do intervalo QT corrigido dos dois grupos foram calculados. Três meses após o tratamento com pressão positiva contínua nas vias aéreas, os registros de eletrocardiograma foram novamente obtidos dos 65 pacientes com apneia obstrutiva do sono grave, e seus valores de dispersão do intervalo QT corrigido foram calculados.

**Resultados:**

Havia 44 pacientes do sexo masculino e 21 do sexo feminino com síndrome de apneia obstrutiva do sono grave. A idade, sexo, índice de massa corporal, saturação inicial, saturação mínima, saturação média e índice de dessaturação foram determinados em ambos os grupos. Os intervalos QT corrigido dos pacientes com apneia obstrutiva do sono (62,48 ± 16,29 ms) foram significativamente maiores (*p* = 0,001) do que os do grupo controle (29,72 ± 6,30 ms). Houve diferenças estatisticamente significativas entre os valores de QT corrigido antes e após o tratamento com pressão positiva contínua nas vias aéreas, com intervalos QT corrigido pré-tratamento de 62,48 ± 16,29 ms e 3 meses pós-tratamento, de 41,42 ± 16,96 ms (*p* = 0,001). Houve uma correlação positiva e significativa entre os períodos de dispersão do intervalo QT corrigido e o índice de apneia-hipopneia e índice de hipopneia em pacientes com apneia obstrutiva do sono (*p* = 0,001; r = 0,71; *p* = 0,001; r = 0,679, respectivamente).

**Conclusão:**

O tratamento com pressão positiva contínua nas vias aéreas reduziu a dispersão do intervalo QT corrigido em pacientes com apneia obstrutiva do sono grave. Além disso, o encurtamento de dispersão do intervalo QT corrigido em pacientes com apneia obstrutiva do sono grave pode reduzir o risco de arritmias e doenças cardiovasculares.

## Introduction

Obstructive sleep apnea (OSA) is characterized by repeated episodes of partial (hypopnea) or full (apnea) obstruction of the upper airway during sleep. These events usually cause a reduction in blood oxygen saturation and generally finish with brief arousals.^1^ According to recent epidemiological studies, the prevalence of OSA increases according to body mass index (BMI), gender, age and apnea-hypopnea index (AHI).^2^ When OSA prevalence is examined at AHI > 15, the rates have been reported as 10–17% in males and 3–9% in females.^2^ The AHI classifies the intensity of OSA and is based on the number of apnea and hypopnea events per hour of sleep per night.^3^ OSA is more frequent in middle-aged male patients and in those with arterial hypertension, heart failure, ischemic heart disease, and stroke.^4^, ^5^ Previous studies have stated the estimated risk of coronary artery disease in OSA patients, especially males, as 37%.^6^

Patients with OSA have an elevated risk of cardiac arrhythmias and sudden death due to increased sympathetic activity and stimulation of the renin–angiotensin–aldosterone system.^7^, ^8^ The evaluation of cardiovascular disease in patients with OSA is an important step.^9^ The QT interval (QT) and QT dispersion (QTd) are Electrocardiogram (ECG) parameters used in the evaluation of myocardial repolarization.^10^ The heterogeneity and dynamics of the QT interval are used to demonstrate enhanced sensitivity to ventricular arrhythmias.^11^ It has been observed that circadian changes depend on concentrations of increased catecholamine in variations and circulation in autonomic tone in the normal heart in QT interval.^12^ The QT interval reflects the homogeneity of repolarization, with extended QT intervals precursors of cardiac repolarization.^13^

The QT corrected interval (QTc) calculates the QT interval in a Heart Rate (HR) at 60 bpm.^13^ It is used to detect an increased risk of arrhythmia.^13^ The QTc is also used as a marker to detect the risk of arrhythmia in patients with impaired left ventricular function.^14^ Increased QTc has been shown to increase the risk of arrhythmia in OSA patients.^15^ The QT dispersion interval (QTd) refers to the difference in the longest QT interval and the shortest QT interval in the ECG. QT corrected interval dispersion (QTcd) refers to the difference in the longest QT corrected interval and the shortest QT corrected interval in the ECG.^15^ QTcd denotes regional variation in the QT interval. The QTd (subtraction of the minimum QT interval from the maximum QT interval) is a parameter proposed as an indicator of the spatial dispersion of the recovery time of the alleged ventricle to distinguish a homogeneous from a non-homogenous myocardium.^16^

Nocturnal continuous positive airway pressure (CPAP) treatment has been shown to decrease cardiovascular risk, especially heart failure associated with sleep apnea.^17^

Therefore, in this prospective longitudinal study, the impact of CPAP treatment on cardiac repolarization was evaluated in patients with severe OSA and an age- and gender-matched control population.

## Methods

### Study population

In accordance with the Helsinki II Declaration, prior to beginning this study, all participants signed an informed consent form provided by the Ethics Committee (approval number 159).

At *α* = 0.05 and *β* = 0.20 levels, test power was defined as 0.80 with sample size as 130 (effect size 0.05).

The study was conducted between July 2015 and February 2016. A total of 240 patients were evaluated. The study was formed of 65 patients receiving CPAP treatment and the control group of 65 patients with AHI < 5. The remaining 110 patients were excluded for reasons including cardiological diseases, diabetes mellitus, hypertension, and rejection or irregular use of CPAP treatment. As a result, a total of 130 subjects were included, comprising 65 in the patient group and 65 in the control group.

The study group consisted of 65 patients referred to the sleep laboratory for clinically suspected OSA. The exclusion criteria were chronic atrial fibrillation, second or third atrioventricular block, all bundle branch blocks, permanent pacemakers, symptoms or signs of congestive heart failure, intraventricular conduction defects, diabetes mellitus, Shy-Drager syndrome pericarditis, valvular heart disease, pulmonary emboli, abnormal thyroid function, cardiomyopathies, pulmonary hypertension, abnormal serum electrolyte values, neurological disease, use of digitalis, use of antiarrhythmic agents, use of betablockers, or use of calcium antagonists, including verapamil and diltiazem, affecting the heart rate (HR). All the patients underwent full polysomnography and 12 lead ECG recordings. Following polysomnography, CPAP pressure titration was applied to the patients (Respiranics, Philips, USA). The diagnosis of OSA was confirmed in all the patients, and they all underwent nocturnal CPAP treatment. Polysomnography was performed, and 12 lead ECG recordings were obtained again 3 months after the commencement of the CPAP treatment. The control group consisted of the ECG recordings of 65 age- and gender-matched individuals, with an AHI of <5, who were free of cardiac disease and had no history of hypertension or diabetes mellitus.

### Sleep study and polysomnography scoring

OSA was diagnosed on the basis of clinical criteria and the results of a 55 channel polysomnograph (Respiranics ALIS 5, Philips, USA). The polysomnography included seven electroencephalograms (F3-A2, C3-A2, O1-A2, F4-A1, C4-A1, O2-A1, and CZA1), right and left electro-oculograms, and one electromyogram of the chin muscles for conventional sleep staging. Respiratory airflow was monitored with a nasal cannula connected to a pressure transducer, thoracic and abdominal respiratory movements were measured with piezoelectric strain gauges, and tracheal sound was measured with a microphone. Arterial oxygen saturation (SaO_2_) was continuously measured using a finger oximeter.

Both automated and hand-scored data analyses were obtained and evaluated. Obstructive hypopnea is defined as a reduction of at least 30% in airflow and this reduction must last at least 10 s, and a fall of 3% in SaO_2_ or accompanying arousal. Apnea is defined as a reduction of at least 90% in airflow amplitude, and respiratory events lasting at least 10 seconds.^18^ The apnea index (AI) was defined as the number of episodes of obstructive apnea per hour of sleep. The AHI was defined as the number of episodes of obstructive apnea and obstructive hypopnea per hour of sleep. The mean SaO_2_ and minimal value recorded during sleep (SaO_2_ min) were used as indices of nocturnal hypoxemia. An AHI of ≥15 was taken as the threshold to identify obstructive sleep-related disorders.

### Analysis of the ECG recordings

At the beginning of the study and 3 months post-CPAP treatment, all the patients underwent 12 channel ECG monitoring. The ECG (Nihon Kohden Cardiofax M, Tokyo, Japan) recordings were taken after the patient had rested for at least 10 min in a supine position, with a paper speed of 50 mm/s. In the ECG, the HR rate, QTc interval and QTcd were calculated.

The QT interval was measured from the beginning of the QRS complex until the end of the T wave. The end of the T wave was determined using the tangential method.^19^ Cases where the end of the T-wave was uncertain, such as in the presence of a low T-wave amplitude or isoelectric irregularity, could not be measured. Only ECGs where six or more QT intervals could be measured in at least three precordial leads were included in the study. The QT interval was measured in two consecutive cardiac cycles, and the average was determined. The calculated QT intervals were corrected according to the HR using Bazett's formula (QTc = QT/√RR in seconds), and the corrected QT interval for each lead (QTc) was calculated.^20^ The QTcd for each patient was determined by calculating the difference between the maximum and minimum QTc interval.

### Statistical analysis

The data are presented as the mean ± standard deviation (SD) or as number (*n*) and percentage (%). The numerical data were first tested for normality. The Student's *t*-test was used for parametric data, and the Mann–Whitney *U*-test was used for nonparametric data for group comparisons. The Wilcoxon signed rank test was used to assess within group differences. Categorical data were analyzed using a Chi-square test or Fisher's exact Chi-square test. Associations between continuous variables were measured using Spearman's correlation coefficients (*r*). All analyses were made using SPSS for Windows, version 18.0 (SPSS Inc., Chicago, IL, USA). A *p*-value of < 0.05 was considered statistically significant.

## Results

A total of 44 males and 21 females were included in this study. The basic characteristics and QTcd of the OSA patients and control subjects are shown in Table 1. There were no significant differences between the OSA patients and the control group with respect to gender, age, and body mass index (BMI). The start saturation, minimum saturation, and average saturation were higher in the control group than in the OSA patients, with significant between-group differences detected (*p* = 0.001). The desaturation index was significantly lower in the control group than in the OSA group (*p* = 0.001). There was no significant difference in the total sleep time of the control and OSA groups (328.09 ± 5:58 min and 311.70 ± 67.77 min, respectively; *p* = 0.2). Likewise, the analysis of stage 1, stage 2, and REM sleep according to percentiles of REM periods revealed no significant between-group differences. However, there was a statistically significant difference between the OSA patients and the control group during stage 3 sleep (8.55 ± 7.00 and 13:56 ± 8:53, respectively; *p* = 0001). The sleep efficiency, maximum HR, minimum HR, and average HR were not statistically significant in either group (*p* = 0.181; *p* = 0.025; *p* = 0.453; and *p* = 0.029, respectively).

**Table 1:** The basic data of the OSA patients and controls (*n* = 65).

	OSAS (*n* = 65)	Control (*n* = 65)	*p*
Age (years)	50.7 ± 11.5	44.7 ± 11.3	0.062
Male (*n*) (%)	44 (67.7)	44 (67.7)	0.106
BMI (Kg/m^2^)	30.50 ± 4.90	29.71 ± 5.58	0.198
Total sleep time (min)	311.70 ± 67.77	328.09 ± 5.58	0.200
Stage 1 (%)	19.8 ± 13.8	15.7 ± 7.9	0.184
Stage 2 (%)	63.0 ± 13.9	60.0 ± 7.6	0.106
Stage 3 (%)	8.5 ± 7.0	13.6 ± 8.5	0.001
REM (%)	8.4 ± 5.8	11.2 ± 7.2	0.014
Sleep efficiency (%)	76.61 ± 12.43	79.02 ± 12.50	0.181
AI (per h)	23.36 ± 24.90	1.32 ± 2.87	0.001
AHI (per h)	35.32 ± 17.93	4.49 ± 3.79	0.001
HI (per h)	6.48 ± 3.79	4.45 ± 3.96	0.001
QTcd time (ms)	62.48 ± 16.29	29.72 ± 6.30	0.001
Maximum heart rate (pulse min^−1^)	123.9 ± 29.2	111.7 ± 18.2	0.025
Minimum heart rate (pulse min^−1^)	54.6 ± 9.5	53.2 ± 6.6	0.453
Mean heart rate (pulse min^−1^)	75.7 ± 12.4	71 ± 8.1	0.029
Initial saturation (%)	93.86 ± 2.13	95.72 ± 1.34	0.001
Minimum saturation (%)	75.11 ± 12.42	89.06 ± 2.84	0.001
Mean saturation (%)	93.05 ± 2.55	95.22 ± 1.76	0.001
Desaturation index (per h)	32.11 ± 24.44	3.54 ± 2.47	0.001

BMI, body mass index; AI, apnea index; AHI, apnea-hypopnea index; HI, hypopnea index; QTcd, QT corrected interval dispersion.

*p*-Values were determined using Chi-Square test for categorical variable, Student *t*-test or Mann–Whitney test for continuous variable (*p* < 0.05).

CPAP (65.6%) was used in 42 patients, auto CPAP (20%) in 13 patients and BiPAP (15.3%) in 10 patients. The mean pressure of patients where CPAP was used was 7.6 ± 1.75 mbar and the mean pressure of patients where BiPAP was used was 14.6/10.3 mbar. A nasal mask was applied to 37 patients and an oronasal mask to 28 patients. The mean PAP treatment was calculated as 6.33 ± 0.98 h/night.

**Figure 1:**
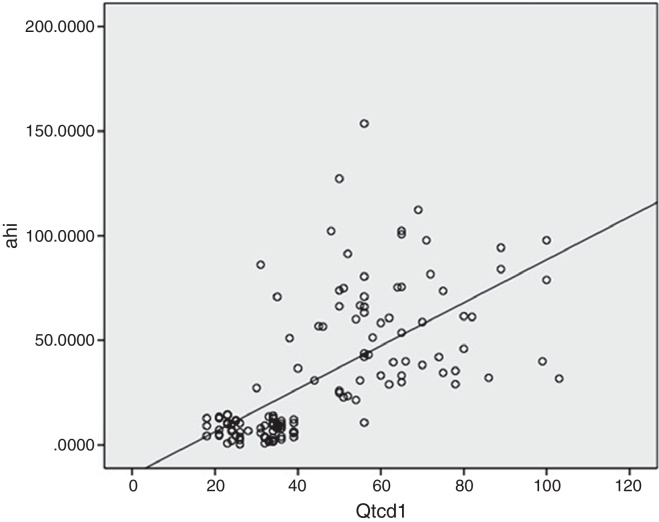
Positive correlation between the QTcd periods and the AHI of the OSA patients. QTcd1, QT corrected interval dispersion in patients with OSAS before CPAP treatment; AHI, apnea-hypopnea index; *p* and *r* values were determined using Spearman correlation *p*: 0.001, *r*: 0.710.

**Figure 2:**
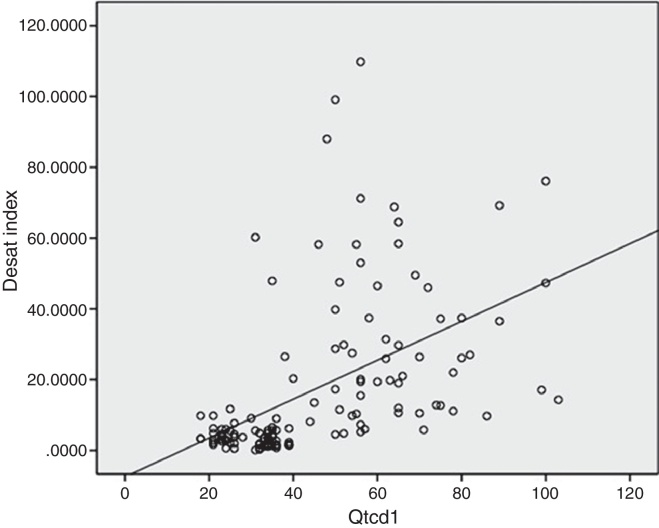
Positive correlation between the QTcd periods and desaturation indices of the OSA patients. Desat index, desaturation index; QTcd1, QT corrected interval dispersion in patients with OSAS before CPAP treatment; *p* and *r* values were determined using Spearman correlation *p*: 0.001, *r*: 0.710; Spearman correlation *p*: 0.001, *r*: 0.654.

The QTcd of the OSA patients was significantly higher than that of the control group (62.48 ± 16.29 ms and 29.72 ± 6.30 ms, respectively; *p* = 0.001). There were also statistically significant differences between the pre-treatment and 3 month post-CPAP treatment QTc values (*p* = 0.001). Furthermore, the pre-treatment QTcd value was longer (62.48 ± 16.29 ms) than the post-treatment QTcd value (41.42 ± 16.96 ms). As shown in Fig. 1, there was a positive and significant correlation between the QTcd periods and the AHI and HI in the patients with OSA (*p* = 0.001, *r* = 0.71; *p* = 0.001, *r* = 0.679, respectively). There was also a positive and significant correlation between the QTcd periods and desaturation indices of the OSA patients (*p* = 0.001, *r* = 0.654) (Fig. 2).

## Discussion

A strong correlation has been determined between OSA and heart failure, hypertension and cardiovascular diseases such as arrhythmia.^21^ With CPAP therapy, the heart rate and diastolic and systolic blood pressures are regulated and at the same time cerebrovascular and cardiological vents have been found to be reduced.^6^ In the 65 OSA patients of the current study with mean AHI 35.32, the QTcd periods were found to be longer than those of the control subjects. After a mean 3 months of CPAP treatment, a reduction in QTcd periods was determined.

In OSA, the individual is unable to breathe due to the collapse of the upper airway during sleep.^22^ Obesity, age, and male gender are the main risk factors.^2^ A higher prevalence of OSA has been determined in those aged 50–70, and it is more common in the male gender.^2^ In the data of population-based studies, OSA has been determined at rates 1.5–3 fold higher in males than females. This ratio in undiagnosed OSA patients has been reported as 2:1.^23^ In our study, the average age of the patients with OSA was 50.72 ± 11.47 years, and 67.7% of the study population were males.

Hypoxia, hypercapnia, autonomic nervous system activation, and negative intrapleural pressure changes occur in OSA. These changes are thought to lead to the deterioration of the baroreceptor response in the autonomic nervous system and to increased mortality, depending on the presence of arrhythmias.^24^

QTd represents regional heterogeneity in myocardial repolarization.^25^ This heterogeneity in the myocardial repolarization time is the result of regional slow down or variations in the transmission path, as illustrated by a delay in the duration of the action potential. In the presence of augmented QTd, the homogeneity of ventricular repolarization declines, thereby increasing ventricular instability.^25^ Non-homogeneous message exchange, displayed with monophasic action potential measurement, but the need for invasive electrophysiological studies is used routinely.^26^ Non-uniform conduction velocity in different parts of the ventricles or the repolarization re-entry mechanism can cause serious ventricular arrhythmias and hence sudden cardiac death.^27^ Research has shown that the QTcd was associated with peripheral vascular disease, ischemic heart disease, dilated and hypertrophic cardiomyopathy, and hypertension in patients with end-stage renal disease and that it increased cardiovascular mortality and morbidity.^28^

QTc measurements are preferred to QTcd when 12 lead simultaneous recordings cannot be obtained because of variations between interleadsor calculation errors.^20^ In the present study, Bazett's index was used to calculate the QTcd.^20^

Extramiana et al. suggested that β-blocker therapy could reduce the QT rate, depending on the daily modulation.^29^ It was also showed that the rate of ventricular repolarization affected the sympathetic nervous system, either directly or indirectly.^29^ Ahnve and Vallin reported that the cholinergic system directly affected ventricular repolarization of the myocardium in 13 healthy volunteers.^30^ Another study reported that autonomic neuropathy was common in OSA patients and that it affected the parasympathetic system at the nerve node level.^31^

In a meta-analysis of OSA patients and those with congestive heart disease, improvements were determined in ejection fraction, diastolic blood pressure and heart rate, with CPAP treatment.^32^

Dursunoğlu et al. showed that the QTcd depended on the intensity of sleep apnea in patients without hypertension and that it was particularly high among those with moderate-to-severe apnea.^10^ In another study, the QTcd declined significantly following CPAP treatment.^15^ In the same study, the QTcd period before CPAP treatment was 54.5 ± 8.7 ms, while at 6 months after treatment, it was found to be 35.5 ± 4.2 ms.^15^ In the current study, the QTcd was evaluated as 62.48 ± 16.29 ms before treatment and as 41.42 ± 16.96 ms after 3 months of CPAP treatment. Using 1231-metaiodobenzylguanidine cardiac imaging, Nakamura et al. found an association between cardiac sympathetic activity and the QTcd in 48 OSA patients.^33^ Çiçek et al. reported that nocturnal QTcd values increased in OSA patients following CPAP therapy independent of a decrease in cardiac sympathetic function.^34^ Roche et al. showed that treatment of patients with severe OSA with CPAP significantly improved their QT dynamics but had no impact on ventricular ectopic activity and static repolarization.^35^ In the current study, in parallel with the literature, the QTcd was shortened following the CPAP therapy.

The impact of the severity of the disease on the HRs of OSA patient's not receiving treatment has not been clarified. The rate of depolarization of the sinoatrial node is determined mainly by the activity of the autonomic nervous system. Thus, the HR is directly dependent on sympathetic activity and autonomic imbalance. A previous study demonstrated that the HRs of OSA patients increased because of activation of the sympathetic system.^34^ Sumi et al. reported a decrease in the number of day and night-time OSA events in patients receiving CPAP therapy three/four times daily.^36^ Kawano et al. reported that increases in the HRs of awake and sleeping OSA patients were independent of the severity of the condition.^37^ They also reported that the average HRs of OSA patients decreased after 6 months of CPAP treatment.^37^ In the current study, no differences were detected in the HRs of the control group and the OSA patients.

In OSA patients, hypoxemia-dependent apnea and hypopnea play an important role in the increased QTcd and HR variability. A previous study has shown that hypoxemia-dependent apnea and hypopnea enhanced the activity of the sympathetic nervous system by stimulating carotid body chemoreceptors.^38^ This indicated an association between arterial oxygen desaturation and arousal due to the over-activation of the sympathetic system.^39^ In a study of the risk of cardiac disease in patients with sleep apnea syndrome, Aydin et al. found a significant correlation between the AHI and P-wave dispersion and an increased risk in patients with severe OSA.^40^ In the present study, a positive correlation was determined between the AHI and HI and QTcd. A positive correlation was also determined between the desaturation index and QTcd.

A limitation of the study was that patients could have been followed up for longer. A comparison of QTcd of patients at 3, 6 and 12 months after CPAP treatment would provide a greater contribution to literature. In addition, that the study was not of a randomized design could also be considered a limitation.

In the present study, the QTcd of the OSA patients decreased following CPAP treatment. A positive correlation was determined between the AHI and desaturation index and QTcd value, which was consistent with findings in literature. However, the mean HR values of the control group did not differ significantly from those of the OSA patients. HR variability in the early period is not among the descending parameters in OSA, which have attracted attention in literature.^41^

## Conclusion

In the present study, the QTcd declined in patients with severe OSA following CPAP treatment. Shortening the QTcd period may reduce the risk of arrhythmias and cardiovascular disease in patients with severe OSA. Studies of the QTcd in patients with sleep apnea who have undergone surgery would contribute to the literature on reducing the risk of cardiovascular disease.

## Conflicts of interest

The authors declare no conflicts of interest.
